# Supplementary material for: EFHC1, implicated in juvenile myoclonic epilepsy, functions at the cilium and synapse to modulate dopamine signaling
Source: eLife. 2019 Feb 27;8:e37271. doi: 10.7554/eLife.37271 (PMC6392500; doi:10.7554/eLife.37271)
Supplement: Supplementary file 1. [file elife-37271-supp1.docx]

**Supplementary File 1**

**Table S1. Sample sizes (n) for tap habituation experiments**

| **Figure** | **Strain** | **Minimum n for a single tap** | **Experimental dates** | **Number of plates** |
| --- | --- | --- | --- | --- |
| 2B | wild-type | 274 | 2013-09-27 | 9 |
|  |  |  | 2013-10-25 | 6 |
|  |  |  | 2013-11-01 | 1 |
|  | *efhc-1(gk424336)* | 440 | 2013-09-27 | 5 |
|  |  |  | 2013-10-25 | 9 |
|  |  |  | 2013-11-01 | 6 |
|  | *dat-1* | 411 | 2013-09-27 | 6 |
|  |  |  | 2013-10-25 | 9 |
|  |  |  | 2013-11-01 | 4 |
|  | *cat-2* | 273 | 2013-09-27 | 5 |
|  |  |  | 2013-10-25 | 8 |
|  |  |  | 2013-11-01 | 4 |
| 2C | wild-type | 284 | 2017-10-02 | 3 |
|  |  |  | 2017-10-03 | 7 |
|  |  |  | 2017-10-23 | 2 |
|  |  |  | 2017-10-24 | 4 |
|  | *efhc-1(gk424336)* | 292 | 2017-10-02 | 4 |
|  |  |  | 2017-10-03 | 6 |
|  |  |  | 2017-10-23 | 2 |
|  |  |  | 2017-10-24 | 4 |
|  | *efhc-1(tm6235)* | 242 | 2017-10-02 | 3 |
|  |  |  | 2017-10-03 | 7 |
|  |  |  | 2017-10-23 | 2 |
|  |  |  | 2017-10-24 | 4 |
| 2D | wild-type | 255 | 2013-12-16 | 3 |
|  |  |  | 2015-05-12 | 7 |
|  | *efhc-1(tm6235)* | 262 | 2013-12-16 | 2 |
|  |  |  | 2015-05-12 | 7 |
|  | *cat-2* | 231 | 2013-12-16 | 3 |
|  |  |  | 2015-05-12 | 8 |
|  | *efhc-1;cat-2* | 173 | 2013-12-16 | 2 |
|  |  |  | 2015-05-12 | 7 |
| 4A | wild-type | 374 | 2013-11-29 | 7 |
|  |  |  | 2015-07-07 | 8 |
|  | *efhc-1(gk424336)* | 382 | 2013-11-29 | 8 |
|  |  |  | 2015-07-07 | 8 |
|  | *trp-4* | 334 | 2013-11-29 | 7 |
|  |  |  | 2015-07-07 | 8 |
|  | *efhc-1;trp-4* | 377 | 2013-11-29 | 9 |
|  |  |  | 2015-07-07 | 8 |
| 4B | wild-type | 252 | 2015-07-21 | 8 |
|  |  |  | 2015-09-02 | 5 |
|  | *efhc-1(gk424336)* | 248 | 2015-07-21 | 8 |
|  |  |  | 2015-09-02 | 5 |
|  | *daf-19* | 123 | 2015-07-21 | 5 |
|  |  |  | 2015-09-02 | 3 |
|  | *efhc-1;daf-19* | 241 | 2015-07-21 | 7 |
|  |  |  | 2015-09-02 | 5 |
| 4C | wild-type | 442 | 2015-09-08 | 7 |
|  |  |  | 2015-09-15 | 8 |
|  |  |  | 2017-10-09 | 2 |
|  |  |  | 2017-10-10 | 4 |
|  |  |  | 2017-10-23 | 2 |
|  |  |  | 2017-10-24 | 4 |
|  | *efhc-1(gk424336)* | 448 | 2015-09-08 | 9 |
|  |  |  | 2015-09-15 | 8 |
|  |  |  | 2017-10-09 | 2 |
|  |  |  | 2017-10-10 | 4 |
|  |  |  | 2017-10-23 | 2 |
|  |  |  | 2017-10-24 | 4 |
|  | *unc-2(gf)* | 495 | 2015-09-08 | 7 |
|  |  |  | 2015-09-15 | 8 |
|  |  |  | 2017-10-09 | 2 |
|  |  |  | 2017-10-10 | 4 |
|  |  |  | 2017-10-23 | 2 |
|  |  |  | 2017-10-24 | 4 |
|  | *pefhc-1::unc-2(gf)* | 459 | 2015-09-08 | 7 |
|  |  |  | 2015-09-15 | 8 |
|  |  |  | 2017-10-09 | 2 |
|  |  |  | 2017-10-10 | 4 |
|  |  |  | 2017-10-23 | 2 |
|  |  |  | 2017-10-24 | 4 |
|  | *efhc-1;pefhc-1::unc-2(gf)* | 372 | 2015-09-08 | 6 |
|  |  |  | 2015-09-15 | 7 |
|  |  |  | 2017-10-09 | 2 |
|  |  |  | 2017-10-10 | 3 |
|  |  |  | 2017-10-23 | 3 |
|  |  |  | 2017-10-24 | 3 |
| Figure 2-figure supplement 2C | wild-type off food | 379 | 2013-09-27 | 8 |
|  |  |  | 2013-10-25 | 8 |
|  |  |  | 2013-11-01 | 10 |
|  | *efhc-1(gk424336)* off food | 247 | 2013-09-27 | 6 |
|  |  |  | 2013-10-25 | 8 |
|  |  |  | 2013-11-01 | 5 |
|  | *dat-1* off food | 153 | 2013-09-27 | 7 |
|  |  |  | 2013-11-01 | 5 |
|  | *cat-2* off food | 242 | 2013-09-27 | 5 |
|  |  |  | 2013-10-25 | 8 |
|  |  |  | 2013-11-01 | 5 |
| Figure 2-figure supplement 2D | wild-type on food | 981 | 2013-09-27 | 17 |
|  |  |  | 2013-10-25 | 14 |
|  |  |  | 2013-11-01 | 11 |
|  | *efhc-1(gk424336)* off food | 494 | 2013-09-27 | 6 |
|  |  |  | 2013-10-25 | 8 |
|  |  |  | 2013-11-01 | 5 |
|  | *dat-1* off food | 306 | 2013-09-27 | 7 |
|  |  |  | 2013-11-01 | 5 |
