# Supplementary material for: EFHC1, implicated in juvenile myoclonic epilepsy, functions at the cilium and synapse to modulate dopamine signaling
Source: eLife. 2019 Feb 27;8:e37271. doi: 10.7554/eLife.37271 (PMC6392500; doi:10.7554/eLife.37271)
Supplement: Supplementary file 2. [file elife-37271-supp2.docx]

**Supplementary File 2**

**Table S2. p values for statistical comparisons**

| **Figure** | **Strain** | **p value (vs.)** |
| --- | --- | --- |
| 2B | wild-type | <1e-10 (*efhc-1*), <1e-10 (*dat-1*),  <1e-10 (*cat-2*) |
|  | *efhc-1(gk424336)* | <1e-10 (wild-type), <1e-10 (*dat-1*),  <1e-10 (*cat-2*) |
|  | *cat-1* | <1e-10 (wild-type), <1e-10 (*efhc-1*),  <1e-10 (*dat-1*) |
|  | *dat-1* | <1e-10 (wild-type), <1e-10 (*efhc-1*),  <1e-10 (*cat-2*) |
| 2C | wild-type | <2e-16 (*efhc-1(gk424336)*), <2e-16 (*efhc-1(tm6235)*) |
|  | *efhc-1(gk424336)* | <2e-16 (wild-type*)*), <2e-16 (*efhc-1(tm6235)*) |
|  | *efhc-1(tm6235)* | <2e-16 (wild-type), <2e-16 (*efhc-1(gk424336)*) |
| 2D | wild-type | <1e-05 (*efhc-1*), <1e-05 (*cat-2*),  <1e-05 (*efhc-1;cat-2*) |
|  | *efhc-1(gk424336)* | <1e-05 (wild-type), <1e-05 (*cat-2*),  <1e-05 (*efhc-1;cat-2*) |
|  | *cat-2* | <1e-05 (wild-type), <1e-05 (*efhc-1*),  0.69 (*efhc-1;cat-2*) |
|  | *efhc-1;cat-2* | <1e-05 (wild-type), <1e-05 (*efhc-1*),  0.69 (*cat-2*) |
| 2E | wild-type | <0.001 (*efhc-1(gk424336)*), 0.040 (*efhc-1(tm6235)*), <0.001 (*dat-1*) |
|  | *efhc-1(gk424336)* | <0.001 (wild-type), 0.115 (*efhc-1(tm6235)*), <0.001 (*dat-1*) |
|  | *efhc-1(tm6235)* | 0.040 (wild-type), 0.115 (*efhc-1(gk424336)*), <0.001 (*dat-1*) |
|  | *dat-1* | <0.001 (wild-type), <0.001 (*efhc-1(gk424336)*), <0.001 (*efhc-1(tm6235)*) |
| 3A  (buzz) | wild-type | 0.00715 (*efhc-1*), 0.01005 (*trp-4*) |
|  | *efhc-1(gk424336)* | 0.00715 (wild-type), 0.84211 (*trp-4*) |
|  | *trp-4* | 0.01005 (wild-type), 0.84211 (*efhc-1*) |
| 3A (press) | wild-type | 0.886 (*efhc-1*), 0.886 (*trp-4*) |
|  | *efhc-1(gk424336)* | 0.886 (wild-type), 1.000 (*trp-4*) |
|  | *trp-4* | 0.886 (wild-type), 1.000 (*efhc-1*) |
| 4A | wild-type | <2e-16 (*efhc-1(gk424336)*), <2e-16 (*trp-4)*), <2e-16 (*efhc-1;trp-4)*) |
|  | *efhc-1(gk424336)* | <2e-16 (wild-type), <2e-16 (*trp-4)*), <2e-16 (*efhc-1;trp-4)*) |
|  | *trp-4* | <2e-16 (wild-type), <2e-16 (*efhc-1(gk424336)*), <2e-16 (*efhc-1;trp-4)*) |
|  | *efhc-1;trp-4* | <2e-16 (wild-type), <2e-16 (*efhc-1(gk424336)*), <2e-16 (*trp-4)*) |
| 4B | wild-type | <2e-16 (*efhc-1(gk424336)*), <2e-16 (*daf-19)*), <2e-16 (*efhc-1;daf-19)*) |
|  | *efhc-1(gk424336)* | <2e-16 (wild-type), <2e-16 (*daf-19)*), <2e-16 (*efhc-1;daf-19)*) |
|  | *daf-19* | <2e-16 (wild-type), <2e-16 (*efhc-1)*), <2e-16 (*efhc-1;daf-19)*) |
|  | *efhc-1;daf-19* | <2e-16 (wild-type), <2e-16 (*efhc-1)*), <2e-16 (*daf-19)*) |
| 4C | wild-type | <1e-09 (*efhc-1(gk424336)*), <1e-09 (*unc-2(gf)*), <1e-09 (*pefhc-1::unc-2(gf)*), <1e-09 (*efhc-1;pefhc-1::unc-2(gf)*) |
|  | *efhc-1(gk424336)* | <1e-09 (wild-type), <1e-09 (*unc-2(gf)*), <1e-09 (*pefhc-1::unc-2(gf)*), <1e-09 (*efhc-1;pefhc-1::unc-2(gf)*) |
|  | *unc-2(gf)* | <1e-09 (wild-type), <1e-09 (*efhc-1(gk424336)*), <1e-09 (*pefhc-1::unc-2(gf)*), <1e-09 (*efhc-1;pefhc-1::unc-2(gf)*) |
|  | *pefhc-1::unc-2(gf)* | <1e-09 (wild-type), <1e-09 (*efhc-1(gk424336)*), <1e-09 (*unc-2(gf)*), <1e-09 (*efhc-1;pefhc-1::unc-2(gf)*) |
|  | *efhc-1;pefhc-1::unc-2(gf)* | <1e-09 (wild-type), <1e-09 (*efhc-1(gk424336)*), <1e-09 (*unc-2(gf)*), <1e-09 (*pefhc-1::unc-2(gf)*) |
| Figure 2-figure supplement 2A | wild-type off food | < 0.05 (wild-type on food) |
|  | *efhc-1(gk424336)* off food | < 0.05 (*efhc-1(gk424336)* on food) |
|  | *cat-2* off food | > 0.05 (*cat-2* on food) |
| Figure 2-figure supplement 2C | wild-type | <2e-16 (*efhc-1*), <2e-16 (*dat-1*),  <2e-16 (*cat-2*) |
|  | *efhc-1(gk424336)* | <2e-16 (wild-type), <2e-16 (*dat-1*),  <2e-16 (*cat-2*) |
|  | *dat-1* | <2e-16 (wild-type), <2e-16 (*efhc-1*),  <2e-16 (*cat-2*) |
|  | *cat-2* | <2e-16 (wild-type), <2e-16 (*efhc-1*),  <2e-16 (*dat-1*) |
| Figure 2-figure supplement 2D | wild-type on food | 0.484 (*efhc-1* off food), <1e-04 (*dat-1* off food) |
|  | *efhc-1(gk424336)* off food | 0.484 (wild-type on food), <1e-04 (*dat-1* off food) |
|  | *dat-1* off food | <1e-04 (wild-type on food), <1e-04 (*efhc-1* off food) |
| Figure 3-figure supplement 1A  (buzz) | Wild-type | 0.943 (*efhc-1(gk424336)*) |
| Figure 3-figure supplement 1A  (press) | Wild-type | 0.597 (*efhc-1(gk424336)*) |
